# Notes on the Epidemiology of Multiple Sclerosis, with Special Reference to Dietary Habits

**DOI:** 10.3390/ijms15033533

**Published:** 2014-02-26

**Authors:** Klaus Lauer

**Affiliations:** Eulerweg 4, D-64347 Griesheim, Germany; E-Mail: drklauslauer@aol.com

**Keywords:** multiple sclerosis, epidemiology, diet, meat, nitrophenols

## Abstract

A hypothesis, based primarily on the occurrence of multiple sclerosis (MS) in the Faroe Islands and supported by numerous analytical epidemiological studies, is described. It proposes that MS is caused by the interaction of a virus disease with intestinal pathology, e.g., infectious mononucleosis, and application of smoked and nitrate/nitrite-cured meat products in the diet during circumscribed time intervals. The biological mechanisms might involve a break of tolerance by an alteration of self within the central nervous system, by nitrophenylated compounds conjugated to animal tissue, in particular to proteins occurring in the central nervous system. Further research is needed.

## 1. Introduction

The aetiology of multiple sclerosis (MS) is still unknown, but both the genetic and environmental fields contribute to the emergence of the disease. Of the environment, in particular, prospective cohort studies revealed in recent years that prior infection with Epstein-Barr virus (EBV) [1–5], deficiency of vitamin D [1,4,6], and tobacco smoking [1,4,7,8] are important for the generation of MS in a multifactorial way. With diet, as one of the possible causes of MS, the situation is at present inconsistent. In many reviewing articles of its epidemiology, diet is also mentioned as a factor of importance [9–12], but e.g., studies mainly devoted to nutrients, and fats in particular, remained controversial [13–16]. Subsequently, processed meat or, more specifically, smoked meat, was outlined [17,18] as the further specification of an earlier hypotheses claiming the role of meat, or pork [19–21] in general. In the present review, the origin of this hypothesis is described in detail, recent ecological findings from Australia are given, and biological mechanisms for the emergence of an autoimmune disease are outlined.

## 2. The Epidemiological Origin of the Hypothesis

In 1979, J.F. Kurzke and K. Hyllested [22] described, for the first time, a unique feature of multiple sclerosis (MS) epidemiology. They reported on MS as an only transitory disease in the Faroe Islands (FI), in the North Atlantic, which are a part of the Kingdom of Denmark. The disease had started there, with the first case ever, in 1943 followed by an epidemic lasting until the early 1950s. Subsequently four minor “outbreaks” of MS followed until 1994 [23–25]. The authors interpreted this pattern as indicating primarily an infectious aetiology of MS, and postulated a hypothetical “primary MS affection (PMSA)” virus, which was introduced by British forces during World War II. In 1989, I took up this epidemiological phenomenon and made an ecological study of diet over time, assembling a large number of books and papers in Danish, Faroese and English languages from the anthropological and socio-cultural human arts [26]. I made also two visits to the FI in 1986 and 1987. The period thus covered reached from *ca.* 1670 up to 1976. The main result was rather surprising: only five dietary factors could be found which occurred only transitorily in the first half of the 20th century, in spite of numerous features which appeared newly over time, but remained present. Four of these factors appeared rather anecdotic or were limited to an insular population, and could not explain the overall epidemiology of MS. The fifth aspect however, was of paramount interest. The population of the FI had traditionally preserved their fish, whale meat and mutton by drying in the sun and the ever blowing wind until the early 20th century [26], as formerly did, in a very similar way, the population of the Shetland Islands [27,28]. In Shetland, however, the habit of drying in the so-called *vivda* (a drying house) felt into complete oblivion, with the emergence of salt handling, until mid 1900 [29,30], and was replaced by salting and smoking [31]. In the FI, however, wind-drying remained the usual method of meat preservation, which was still alive in 1986 ([29]; own observation in 1986). However, in 1907, there was a severe interruption in form of the first cookery book in Faroese language, published by Helena Patturson in Tórshavn, Faroe Islands, in which she recommended the smoking of meat and fish and the use of “wood vinegar” (a distillation product of natural wood, which was rather common at that time in the Scandinavian countries, as it is now in many parts of the world) for meat preservation [32]. Later remarks in the literature from the 1930s to early 1950s confirmed this behavior [33–36], until electric appliances appeared in the kitchen in the 1950s and 1960s, and the usual peat-burning stoves, used for cooking and meat smoking before, disappeared within a decade [36,37]. The Faroese, however, who were always in conflict with the “strange” method of smoking for preservation, took again quickly the old form of wind-drying, which is still present today ([29]; own observation in 1986). However, another trait came into play from the mid 1960s on, *i.e.*, the import of pork and sausages, mostly from Denmark, which finally amounted to a considerable part of meat products [37]. This situation remained until the present day, with supermarkets located in the larger towns of the FI that provide both local and imported foods (own observation in 1986 and 1987). The MS incidence was shown, in 2011, by P. Joensen to have risen again, after a transitory decline from 1963–1982, up to 4.9 per 100,000 per year in 1993–2002, and the MS prevalence amounted to 95 per 100,000 in 2011 [38]. Joensen, however, in contrast to Kurtzke and Hyllested 1979 [22], found the first case of MS in the FI with onset of his disease in 1917, and the second in 1939 [38], followed by the “waxing-and-waning” curve which had already been shown by the former authors [22–25] and representing the first epidemic of MS in the FI.

## 3. Further Epidemiological Findings in Support of the Hypothesis

In the following years, the method of meat preservation was studied in, so far, 14 regions, which have a number of sub-regions sufficiently large to make an ecological investigation. In nearly all of these regions, a statistically significant association could be shown with either the MS prevalence, or the MS mortality, or the regional “case-control ratio” as an indicator of MS prevalence, ([39–49]; Table 1). Only in Australia, including seven territories, did a more complex pattern emerge [50]: whereas the MS prevalence based on data from the MS society 2010 [51], as compared with diet in 1995 [52], indicated an even “protective” role of processed meat (*n* = 7; *r*_S_ = −0.739; *p* = 0.058), data from the Survey of Disability, Ageing and Carers (SDAC) of the Australian Bureau of Statistics [51] revealed exactly the reverse (*n* = 7; *r*_S_ = +0.775; *p* = 0.041; [50], Table 1). Since this data source included only the more disabled patients of MS, it might be speculated that processed food causes a more severe course of MS rather than the risk *per se*. Further studies are needed.

In Scandinavia [40] and Canada [44], coastal areas were also included, and these showed either no difference from the mean or even a lower MS rate. This fact gave rise to the further sub-hypothesis that some factor(s) occurring specifically in fish and seafood, which is traditionally more prevalent in the local diet in coastal regions, are rather more protective from MS. This was recently confirmed by D’hooge *et al*. [53] who showed a less severe course of MS among patients with the relapsing—remitting type of MS who eat more fish.

The individual risk of MS was also studied, so far, in six case-control studies ([54–59]; Table 2). With that study type, in which a much higher level of evidence can be reached, significant results were also found in all regions, implicating a higher risk for smoked meat. In two of these studies, the search for risk factors was restricted to a particular class of so-called “hot-smoked” sausages, *i.e.*, frankfurters, Vienna sausages, or “hot dogs”. It should be mentioned that smoking was the usual method of meat preservation, in temperate regions of Europe, since several thousand years of human history [60], *i.e.*, long before MS appeared around 1800 [61,62]. This is in contrast to another factor, which is linked to smoking, *i.e.*, the pre-curing with nitrate, or nitrite. In Germany, for example, it was introduced e.g., *ca.* 1750, but it took a large up-swing after the detection of saltpetre mines in Chile in the 19th century [63]. The temporal pattern of its usage is very similar to the emergence of MS in the medical literature [61,62].

The occurrence of MS in some religious groups gave also support to the present hypothesis [64]. These Anabaptist groups have their background in the haunting German history of the late 16th century. One community, the Hutterites who are of Southern German and Austrian descent, but had finally, after a long migration mainly through Moravia, Romania and the Ukraine, immigrated to southern Canada and the northern USA in the late 1800s, have a surprisingly low rate of MS [65,66]. Mennonites, in contrast, which are of north German and Dutch descent and also immigrated to southern Canada, and later partly to Latin America, seem to suffer from MS at least as common as their Canadian ethnic neighbors [67]. The Hutterites who still practice communal property have, until the present day, the habit to preserve all their food in large refrigerated storing facilities ([68,69]; own observation in 1998), which is in line with the prescribed avoidance of any pleasure in daily food. Old Colony Mennonites, in contrast, who rigidly observe their traditional faith, smoke and cure much of their processed meat with nitrites [70,71].

Another example of a low rate of MS among Whites is presented by immigrants to South Africa [72]. In that country, where the climate is comparatively warm and dry, *biltong* and *droewors* play a major role in the diet, and both products are cured with nitrite, but dried in the air [73,74]. This pattern indicates that only the synthesis of both preservation methods, *i.e.*, curing with nitrites and subsequent smoking, is the center of the present hypothesis.

The question of an association was also investigated in a single large epidemiological cohort study in the USA [75]. In that investigation, no risk was found for processed meat, 80% of which are smoked in developed countries. However, only the year preceding the onset of MS, and not childhood and early adolescence relevant to MS emergence [76,77], were studied for exposure. Thus, the hypothesis remains open at present. As a possible alternative, results of an extended case-control study of MS in five regions of the globe, the EnvIMS study, which goes on in four European countries and in Canada [78] and which is including also food preservation methods in detail, must be awaited.

## 4. Biological Mechanisms

The fact that only smoked meat, but not smoked fish, showed an almost universal relation to MS led to the further sub-hypothesis that (an) additional factor(s) peculiar to meat, but not to fish, interacts with smoking (*vide supra*). One factor meeting this criterion is prior curing, which today is commonly made by a mixture of cooking salt and nitrites, which is applied before smoking. Nowadays, in the era of refrigerators and freezers, the main purpose of curing is to preserve the red color of the meat, by allowing chemical reactions with myoglobin [79], but also an anti-botulinum activity of nitrites plays a role [80]. Fish, in contrast, is only rarely cured with nitrates, or nitrites, but exceptions occur, e.g., in Scotland [81]. In 1975, Knowles *et al*. [82] described the formation of different nitrophenol compounds in raw and fried cured, smoked bacon. Their findings were later confirmed in Germany, where even dinitrophenols were found [83]. Some years earlier, the conjugation of smoke phenols, via lysine residues, to meat proteins was reported [84] and, with all the likelihood, this conjugation also occurs with nitrophenols. Unfortunately, however, no quantitative data are available until the present day. Already in 1962, W.O. Weigle [85,86] had experimentally demonstrated the breaking of immune tolerance towards thyroglobulin, by altering the antigen-moiety by trinitrophenol (TNP)—binding of that protein. Remarkably, thyreoiditis developed in two out of six surviving animals [86], which was probably autoimmune in character. In later reports from the 1990s, Martin *et al*., and Martin and Weltzien investigated the consequences of nitrophenolation of autoantigens for T cell recognition, in the context of accommodation to the binding grooves of the major histocompatibility complex (MHC) molecules. They observed (a) the alteration of the framework, in contrast to the variable, parts of the T cell receptor by the trinitrophenylated antigen, which is unusual and might be a reason for the large spectrum of changed specificities; and (b) that hydrophobic, in particular phenolic, and heterocyclic amino acids played a major role in the binding contact [87–89]. The authors had devoted their studies to drug allergies, but the outstanding results may be generalized and applied to a broader range of conditions, including autoimmunity.

Furthermore, Achtnich and Zoeller demonstrated, in mice, the later development of a generalized autoimmune condition, with polyspecific antibodies to several autoantigens and TNP, when the reactive compound trinitrobenzensulphonic acid (TNBS) was applied very early to the animals [90]. It is very likely that this low-weight compound, which is well known as a hapten, subsequently conjugates with different mouse proteins as carriers and, thus, causes autoimmunity. However the present hypothesis would still be more convincing and consistent with Weigle’s data on autoimmune thyreoiditis, when animal proteins of the central nervous system (CNS) and conjugated as carriers *ex vivo* were applied to the mucosal immune system of the gut via daily diet. This might be especially occurring in transitory time periods when the intestine is in an abnormal state of less stringent barriers, e.g., during a variety of gut infections [91]. A survey of brain antigens that are present in processed meats revealed that 9.9% of sausage samples were reacting positively with proteolipid protein (PLP). This protein is practically specific to the CNS and proves prior usage of brain material during the preparation of sausage [92]. It had not been declared to the consumer by labeling, as prescribed by the European Union, even when non-cattle and non-ovine (e.g., swine) brain and spinal cord material is added. Earlier studies, using a different methodology with cholesterol screening and neuron-specific enolase (NSE) as markers of CNS revealed 4% positivity [93,94]. In a later publication, with use of polyclonal anti PLP-antibody, a lower percentage of positive results (*ca*. 1%–2%) were found [95], which were ascribed to the new BSE crisis at that time. The usage of CNS material in sausage preparation is of some advantage to producers due to its emulgating properties and its low price [93]. In Germany, however, the admixture of brain and spinal cord from cattle and sheep from age 12 months is now prohibited, and meat products need a special declaration if they are made with CNS material e.g., from swine regardless of age or from cattle younger than 12 months [92].

In many countries, it is now common to use commercial smoke distillation preparations (e.g., formerly called “wood vinegar”) for treating meat and sausages instead of applying natural smoke for preservation [60,96]. The composition of these products is varying by brand, but a common advantage is the removal of potentially cancerogenic PAHs and tarry constituents. Furthermore the application of liquid smoke, through mixing under the sausage batter or injecting into the meat, ensures a homogeneous flavor of the product [96]. However, their content of e.g., phenols is in same range or even higher than in the natural glowing smoke [60], and the possible consequences for the present hypothesis might even be accentuated due to their higher concentration also in the inner parts of the product.

The sharp discrepancy between the frequent ingestion of smoked meat and sausages on one hand in developed countries, and the comparatively low frequency of MS on the other, suggest that at least one additional, or interacting, factor plays an important role, e.g., infections of the intestine. This assumption is all the more prudent as the mucosal barrier which is rather close under normal conditions, is transitorily disturbed during the period of infection [91], and a more direct link to the core immune system occurs.

The impact of infections, as one possible factor during late childhood and adolescence, is widely discussed since decades in the pathogenesis of MS [1–5]. In particular, infectious mononucleosis by EBV infection causes inflammatory intestinal alterations in a high percentage of cases [97]. A possible synthesis, however, with other environmental factors was only rarely addressed. We had found such an interaction of cured, hot-smoked sausages and common childhood infections (including EBV infection) occurring after age 9 in a case-control study of MS patients and hospital controls [58], but more extended studies are needed.

Another hypothesis might be developed, based upon studies by Berneman *et al*. [98] on natural auto-reactive antibodies, which exist in all healthy individuals [99,100]. They demonstrated the cross-reacting affinity, among many other autoantigens that were reacting with natural antibodies, of myelin basic protein (MBP) (which is one of the central autoantigens discussed in MS [101]) with TNP. One might speculate that prior repeated stimulations with nitrophenol in food (*vide supra*) paved the way to a permanent anti-MBP autoreactivity. Some support was found in the experimental work by Achtnich and Zoeller already discussed who showed that the prenatal application of TNP in form of TNBS acid gave rise to increased later autoimmunity to thyroglobulin, myoglobin, single- and double-stranded DNA in adulthood [90].

A further sub-hypothesis is based on the observation of nitro-polyaromatic hydrocarbons (nitro-PAHs, or nitro-arenes), which occur especially in smoked sausages, less so in other smoked meat [102,103]. Only sparse data are available in the literature on the consequences to the immune system. Studies in diesel exhaust (which, however, contain these compounds in still much larger amounts) have shown that both immunostimulating and immunosuppressive processes, comprising numerous compounds, occur after exposure [104]. Studies on food are not available. However, the high concentrations of nitro-PAHs also found in vegetable food sources [102] argue, at first, against a major role of these compounds. Furthermore, this view is strengthened by the occurrence of MS in the FI, since liquid smoke occurring at that time, is almost lacking the PAHs, but nonetheless was related to a sharp increase of the MS incidence (*vide supra*).

## 5. Conclusions

Some hypotheses can be delineated, based on published data, for the environmental factor “cured and smoked meat”, but if, and to what extent, these play some role in the pathogenesis of MS must await further investigations. Epidemiological evidence is arguing for a role of processed meat and sausages in MS. Research related to the food industry and artisan’s operations has elaborated alternatives to nitrite in meat curing [80], but commercial considerations and arguments of flavoring are still under discussion. An answer, however, is urgently needed, since western foods, including hot-dogs, appear with rapidly increasing frequency in developing countries. For example, whereas all the Arab countries had a low rate of MS before 1980 [105], the increase of the MS prevalence, from 10 to 85 per 100,000, that was observed in Kuwait within only 30 years [106], is an alarming sign. In Kuwait, there was, after 1991, a large increase of hot-dog consumption which seemed to have changed considerably the local eating habits [107]. In the United Arab Emirates (UAE), even camel-meat hot-dogs are now offered in Dubai [108], where the MS prevalence was in the medium range of 54.8 per 100,000 in 2007 [109]. In the neighboring Abu Dhabi, however, where the MS prevalence was 20.5 per 100,000 in 2003 [110], a retreat has recently been beaten by banning, since 2010, all hot dogs and burgers from public schools as a result of a scientific workshop [111]. Consequently, the present hypothesis could find considerable support, or may be disproved, by the further observation of MS in that country in the next 30 years. An only transitorily frequent occurrence in Abu Dhabi, which was formerly demonstrated only in the FI [22–24,38], would be a strongly supporting argument for the present hypothesis.

## Figures and Tables

**Table 1.** Correlation of MS prevalence, or MS mortality, with processed, or smoked, meat intake, favouring a pathogenetic role, in 14 regions.

Region	Subunits	MS Rate	*p*-Level	References
France	12 regions	mortality	0.020	[39]
Switzerland	22 cantons	prevalence	0.002	[39]
Sweden	24 provinces	prevalence	0.002	[40]
Norway	18 counties	mortality	<0.001	[40]
Finland	9 counties	prevalence	0.038	[40]
globe	26 countries	prevalence	<0.001	[41]
USA	48 States	case-control ratio	<0.001	[42]
Lower Saxony, Germany	2 regions	mortality	<0.002	[43]
Canada	10 provinces	mortality	0.001	[44]
European Union	7 countries	prevalence	0.042	[45]
Austria	9 Fed. States	mortality	0.020	[46]
England/Scotland	8 Statist. Regions	hospital admission rate	0.007	[47]
Spain	46 provinces	mortality	0.068	[48]
globe	69 countries	prevalence	<0.001	[49]
Australia	7 territories	SDAC *. data	0.041	[50]

* SDAC: Survey of Disability, Ageing and Carers of the Australian Bureau of Statistics, 2009. Spearman’s coefficient or Mann-Whitney test were applied.

**Table 2.** Case-control studies of processed meats in the MS generation.

Region	OR	*p*-Value	References
South Hesse, Germany	18.5 *	0.004	[54]
Gorski Kotar, Croatia	20.7	<0.001	[55]
Moscow, Russia	17.5	<0.001	[56]
Montreal, Canada	1.2 **	0.030	[57]
South Hesse, Germany	2.8 *	0.001	[58]
Čabar, Croatia	10.8 ***	0.004	[59]

OR: odds ratio;

* only hot—smoked sausages (frankfurters; hot-dogs; *etc*.);

** summary variable of pork and hot-dogs;

*** own calculation from the reverse.
